# Insights into binding events of GABA- and Tiagabine- analogues in the Gamma-Aminobutyric Acid Transporter 1 by means of Molecular Modelling

**DOI:** 10.1186/1758-2946-4-S1-P54

**Published:** 2012-05-01

**Authors:** Barbara Zdrazil, Andreas Jurik, Regina Reicherstorfer, Thomas Stockner, Harald H Sitte, Gerhard F Ecker

**Affiliations:** 1 University of Vienna, Department of Medicinal Chemistry, Pharmacoinformatics Research Group, Althanstrasse 14, A-1090 Vienna, Austria; 2 Medical University Vienna, Institute of Pharmacology, Währinger Straße 13A, A- 1090 Vienna, Austria

## 

The human transporters for the inhibitory neurotransmitter gamma-aminobutyric acid (GABA) hGAT-1, 2, and 3, and hBGT-1 belong to the neurotransmitter-sodium symporter (NSS) family of membrane transport proteins. hGAT-1 has been a target for the design of antiepileptic therapeutics [1], with tiagabine (Gabitril®) being the only GAT inhibitor on the market. The lack of specific inhibitors for the other hGAT subtypes, results from a still missing detailed understanding of the molecular basis of drug-transporter interactions of the respective subtypes.

We aim at elucidating plausible binding modes for ligands of each subtype, respectively. In our first studies, we built up homology models for hGAT-1 in the occluded and outward-facing conformation based on the respective high resolution structures of the leucine transporter of Aquifex aeolicus (LeuT) (pdb-codes: 2A65 and 3F3A). Afterwards, the natural substrate GABA was docked into the occluded state model and tiagabine into the outward-facing model by making use of the Induced Fit Docking module of Schrödinger, LLC. Both models were further subject to extensive Molecular Dynamics (MD) simulation studies (GROMACS 4.5.3 was used).

By the aid of MD simulations we could detect the existence of conserved water molecules into the GABA (occluded) and tiagabine (open-to-out) binding sites, respectively. Average structures from the equilibrated trajectories were extracted and subsequently served for further docking experiments.

Docking of small ligands (GABA, Guvacine and R-/S Nipecotic Acid) into the occluded state model nicely indicated their preference to bind in an extended conformation (also demonstrated by long-term MD with GABA). MD and Docking of tiagabine and analogues into the open-to-out state model revealed a common binding mode.

Additionally, our studies will be extended to the other hGAT subtypes so as to elucidate the secret of subtype selectivity of GABA Transporters.
